# Expression of *Arabidopsis* Hexokinase in Citrus Guard Cells Controls Stomatal Aperture and Reduces Transpiration

**DOI:** 10.3389/fpls.2015.01114

**Published:** 2015-12-16

**Authors:** Nitsan Lugassi, Gilor Kelly, Lena Fidel, Yossi Yaniv, Ziv Attia, Asher Levi, Victor Alchanatis, Menachem Moshelion, Eran Raveh, Nir Carmi, David Granot

**Affiliations:** ^1^Institute of Plant Sciences, Agricultural Research Organization, The Volcani CenterBet Dagan, Israel; ^2^The Robert H. Smith Institute of Plant Sciences and Genetics in Agriculture, The Robert H. Smith Faculty of Agriculture, Food and Environment, The Hebrew University of JerusalemRehovot, Israel; ^3^Institute of Agricultural Engineering, Agricultural Research Organization, The Volcani CenterBet Dagan, Israel; ^4^Department of Fruit Tree Sciences, Institute of Plant Sciences, Agricultural Research Organization, Gilat Research CenterNegev, Israel

**Keywords:** sugar, hexokinase, stomata, transpiration, water-use efficiency, citrus rooted leaves, humidity, light intensity

## Abstract

Hexokinase (HXK) is a sugar-phosphorylating enzyme involved in sugar-sensing. It has recently been shown that HXK in guard cells mediates stomatal closure and coordinates photosynthesis with transpiration in the annual species tomato and *Arabidopsis*. To examine the role of HXK in the control of the stomatal movement of perennial plants, we generated citrus plants that express *Arabidopsis* HXK1 (*AtHXK1*) under KST1, a guard cell-specific promoter. The expression of KST1 in the guard cells of citrus plants has been verified using GFP as a reporter gene. The expression of *AtHXK1* in the guard cells of citrus reduced stomatal conductance and transpiration with no negative effect on the rate of photosynthesis, leading to increased water-use efficiency. The effects of light intensity and humidity on stomatal behavior were examined in rooted leaves of the citrus plants. The optimal intensity of photosynthetically active radiation and lower humidity enhanced stomatal closure of *AtHXK1*-expressing leaves, supporting the role of sugar in the regulation of citrus stomata. These results suggest that HXK coordinates photosynthesis and transpiration and stimulates stomatal closure not only in annual species, but also in perennial species.

## Introduction

Stomata, formed by two guard cells, open at dawn to allow the atmospheric carbon dioxide (CO_2_) needed for photosynthesis to enter the leaf, at the cost of extensive transpirational water loss. When carbon fixation and utilization are less efficient, the stomata close to reduce the loss of water via transpiration (Assmann, 1993). Mechanistically, stomata open in response to increases in the osmolarity of the guard cells. These increases are followed by the movement of water into the guard cells, which opens the stomata (Taiz and Zeiger, 1998). Stomata close when the osmolarity of the guard cells is reduced and the water exits the guard cells.

At the start of the previous century, the prevailing paradigm was that sugars generated from starch degradation in guard cells at dawn are the primary osmolytes that open stomata (Lloyd, 1908). The discovery that K^+^ ions, Cl^-^ ions and malate ions are the primary osmolytes that open stomata (Schroeder et al., 2001; Roelfsema and Hedrich, 2005; Pandey et al., 2007) yielded a modified hypothesis suggesting that K^+^ ions open stomata at dawn and that sugars generated from starch degradation, photosynthetic carbon fixation or the import of apoplastic (intercellular) sucrose replace K^+^ ions over the course of the day and keep stomata open (Gotow et al., 1988; Tallman and Zeiger, 1988; Poffenroth et al., 1992; Talbott and Zeiger, 1993, 1996, 1998; Amodeo et al., 1996). A non-osmotic role for sugars in stomatal opening has been recently suggested. In the suggested scenario, sucrose is cleaved within guard cells by either sucrose synthase or invertase and this cleavage provides substrates for organic acid synthesis and respiration that open stomata (Antunes et al., 2012; Daloso et al., 2015a,b). Yet, recent studies in *Arabidopsis*, tomato and *Vicia faba* have shown that sugars close stomata (Kelly et al., 2013; Li et al., 2015).

Sugar is produced primarily in leaf mesophyll cells. In many plant species, sucrose - a glucose-fructose disaccharide – is the primary transported sugar exported to the intercellular space, the apoplast, prior to being loaded into the phloem (Rennie and Turgeon, 2009). Some of this apoplastic sucrose is carried toward the open stomata by the transpiration stream, so that the concentration of sucrose in the guard cells’ apoplast may reach 150 mM (Lu et al., 1995, 1997; Ewert et al., 2000; Outlaw and De Vlieghere-He, 2001; Kang et al., 2007). It has been suggested that this accumulation of sucrose decreases stomatal apertures due to an extracellular osmotic effect (Outlaw, 2003; Kang et al., 2007). However, recent studies with tomato and *Arabidopsis* have shown that sucrose stimulates stomatal closure independent of its osmotic effect and that this closure is mediated by HXK within the guard cells (Kelly et al., 2013).

Hexokinase is an essential enzyme that phosphorylates glucose and fructose, the products of sucrose cleavage (Dennis and Blakeley, 2000). In plants, HXK is the only enzyme that can phosphorylate glucose and may also phosphorylate fructose (Granot, 2007, 2008). Most studies of HXK in plants have involved *Arabidopsis HXK1* (*AtHXK1*), which mediates sugar-sensing, in addition to its catalytic hexose-phosphorylation activity (Moore et al., 2003; Rolland et al., 2006). In a recent study, we found that HXK mediates stomatal closure in response to sugar levels (Kelly et al., 2013). Furthermore, expression of *AtHXK1* specifically in the guard cells of the annual species tomato and *Arabidopsis*, under the potato-derived KST1 guard cell-specific promoter, stimulated stomatal closure and reduced stomatal conductance and transpiration (Kelly et al., 2013). Yet, the role of HXK in the stomata of perennial species, including trees, is not known. In the current study, we examined the use of KST1 promoter to drive guard cell-specific expression of *AtHXK1* in citrus plants and the effect of HXK on citrus stomatal movement.

## Materials and Methods

### Plant Material and Growth Conditions

Experiments were conducted on the citrus Troyer citrange (*Citrus sinensis* ‘Washington’ sweet orange × *Poncirus trifoliata*). Troyer citrange explants were transformed with the *KSTpro::GFP* and *KSTpro::HXK1* constructs described in (Kelly et al., 2013) using agrobacterium-mediated transformation as described in (Luth and Moore, 1999). Regenerant plants were grafted onto new Troyer citrange rootstock and the graft points were wrapped in polyethylene. After several weeks, the polyethylene was removed and PCR was used to test each plant for the presence of the transgene. Plants transformed with *KSTpro::GFP* or *KSTpro::HXK1* are referred to as GCGFP and GCHXK, respectively. The transgenic plants were vegetatively propagated by grafting shoots onto Troyer citrange rootstocks. The grafted plants were grown in a temperature-controlled greenhouse under natural growth conditions.

### Rooting Leaves

Detached mature leaves (about 2 months old) of WT and GCHXK plants were rooted in a high-humidity polystyrene chamber filled with perlite. The leaves generated roots from the edge of the petiole after approximately 45 days in perlite (**Figures 5A,B**). The rooted leaves were planted in 50-ml Falcon tubes filled with perlite and each tube was covered with Parafilm to prevent evaporation. WT and GCHXK rooted leaves were placed in a growth chamber that was kept at 25°C, with a 12-h light/12-h dark photoperiod (light turned on at 6:00 a.m. and turned off at 6:00 p.m.), humidity set at 35% and one of the following light intensities: 100, 400, 600, and 800 μmol m^-2^ s^-1^ of PAR (photosynthetic active radiation); comprised of 87.5% red light and 12.5% blue light (Led Lamp 300w, N.B. Advanced Solutions).

### Confocal Microscopy Imaging

Images were acquired using the Olympus IX 81 inverted laser scanning confocal microscope (Fluoview 500) equipped with a 488-nm argon ion laser and a 60 × 1.0 numerical aperture PlanApo water immersion objective. Green fluorescent protein was excited by 488-nm light and the emission was collected using a BA 505–525 filter. A BA 660 IF emission filter was used to observe chlorophyll autofluorescence. Confocal optical sections were obtained in 0.5- to l-μm increments. The images were color-coded green for GFP and magenta for chlorophyll autofluorescence.

### RNA Extraction, cDNA Preparation and Quantitative Real-Time PCR

Leaf tissue was harvested from WT and GCHXK plants and total RNA was extracted from that tissue using the Logspin method (Yaffe et al., 2012). In brief, samples were ground using a Geno/grinder (SPEX SamplePrep, Metuchen, NJ, USA) and RNA was extracted in 8 M guanidine hydrochloride buffer (Duchefa Biochemie) and transferred to tubes containing 96% EtOH (Bio Lab, Jerusalem, Israel). Then, samples were transferred through a plasmid DNA extraction column (RBC Bioscience, New Taipei City, Taiwan), followed by two washes in 3 M Na-acetate (BDH Chemicals, Mumbai, India) and two washes in 75% EtOH, and eluted with DEPC (diethylpyrocarbonate) water (Biological Industries, Co., Beit Haemek, Israel) that had been preheated to 65°C. The RNA was treated with RQ1-DNase (ProMega, Madison, WI, USA) according to the manufacturer’s instructions, to degrade any residual DNA. For the preparation of cDNA, total RNA (1 μg) was taken for reverse transcription-PCR using qScript^TM^ cDNA Synthesis Kit (Quanta BioSciences, Gaithersburg, MD, USA) following the manufacturer’s instructions. cDNA samples were diluted 1:7 in double-distilled water. Quantitative real-time PCR reactions were performed using SYBR Green mix (Thermo-Scientific, Waltham, MA, USA) and reactions were run in a RotorGene 6000 cycler (Corbett, Mortlake, NSW, Australia). Following an initial pre-heating step at 95°C for 15 min, there were 40 cycles of amplification each consisting of 10 s at 95°C, 15 s at 55°C, 10 s at 60°C and 20 s at 72°C. Results were analyzed using the RotorGene software. Data were normalized using *Citrus sinensis* actin as a reference gene (accession no. XM_006464503). The following primers were used for amplification:

Actin_F: GTC TGG TCC ATC CAT TGT CCA
Actin_R: CAA TGG CCC CAA CCT TAG C
HK_F: GCC TTT GAA GAG GAT TGT GC
HK_R: CAT GAC ACG GAA GTT TGT CC

### Gas-Exchange Measurements

Citrus stomatal conductance (*g*_s_), photosynthesis and transpiration rates were measured on fully developed leaves of plants grown in a greenhouse, using a Li-6400 portable gas-exchange system (LI-COR, Lincoln, NE). All measurements were conducted between 9:00 a.m. and 12:00 p.m. Photosynthesis was induced under optimal light (600 μmol m^-2^ s^-1^) with 400 μmol mol^-1^ CO_2_ surrounding the leaf (Ca). The amount of blue light was set to 12.5% photosynthetically active photon flux density to optimize stomatal aperture. The leaf-to-air VPD (vapor pressure difference) was kept at around 1.3–1.63 kPa during all measurements. Leaf temperature for all measurements was approximately 25°C (ambient temperature).

### Whole-Plant Relative Transpiration and Continuous Transpiration Rate Measurements

Whole-plant transpiration rates were determined using lysimeters, as described in detail in Sade et al. (2010). Wild-type plants and GCHXK transgenic plants grafted on WT rootstocks were planted in 3.9-L pots and grown under controlled conditions. Each pot was placed on a temperature-compensated load cell with digital output and was sealed to prevent evaporation from the surface of the growth medium. A wet vertical wick made of 0.14-m^2^ cotton fibers partially submerged in a 1-L water tank was placed on a similar load cell and used as a reference for the temporal variations in the potential transpiration rate. The output of the load cells was monitored every 10 s and the average readings over 3 min were logged in a data-logger for further analysis. Whole-plant transpiration was calculated as a numerical derivative of the load cell output following a data-smoothing process (Sade et al., 2010). At the time of the experiment (sunrise was around 6:20 a.m. and sunset was around 7:00 p.m.). The plant’s daily transpiration rate was normalized to the total leaf area (measured using LI-COR area meter model Li-3100) and the data for a neighboring submerged wick and these figures were averaged for each line (amount taken up by the wick daily = 100%).

## Results

### Expression Analysis of the KST1 Promoter in Citrus Plants and Generation of *KST1pro::HXK1* Plants

**FIGURE 1:**
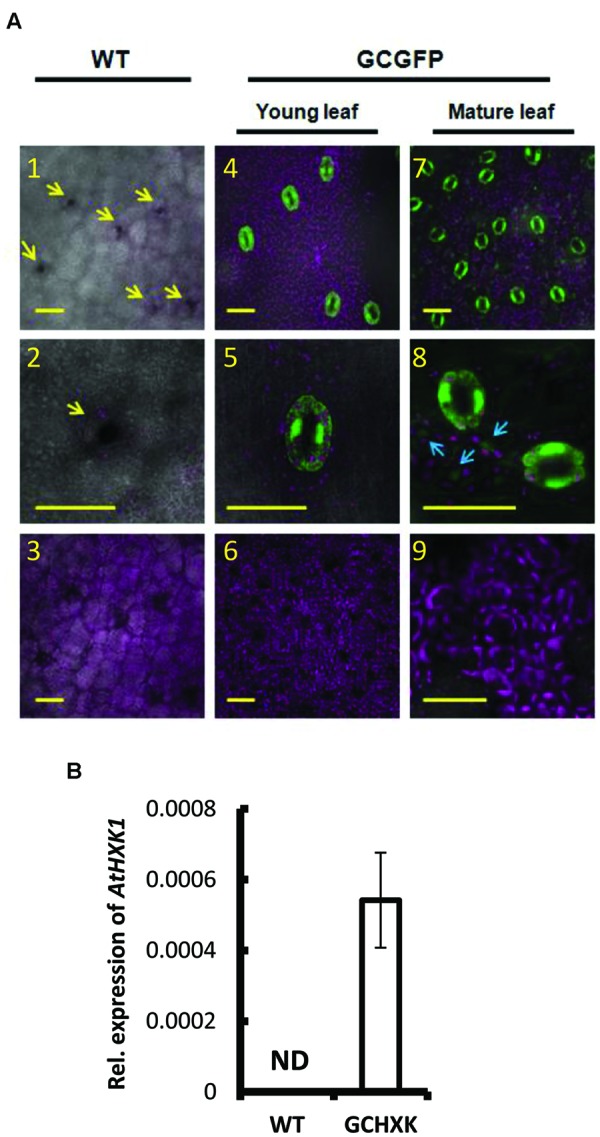
**Expression pattern of KST1::GFP in citrus and expression level AtHXK1 in KST::HXK1 lines. (A)** Confocal images of leaves from wild-type citrus plants (WT; Panels 1–3) and from transgenic citrus plants (Panels 4–9) expressing GFP (designated GCGFP) under the control of the KST1 promoter (*KSTpro::GFP*). Panels 4–6 show young leaves (i.e., less than 1 month old); panels 7–9 show mature leaves (i.e., more than 1 month old). GFP fluorescence stained green and chlorophyll autofluorescence stained magenta. All images are merged with white light images. Panels 1, 4, and 7 show the epidermis level (with a small fraction of the mesophyll level underneath it). Panels 2, 5, and 8 show the enlarged epidermis region and panels 3, 6, and 9 show the mesophyll level. The arrows in panel 1 point toward the stomata and the arrows in panel 8 point toward GFP expression in epidermal pavement cells. Scale bars = 30 μM. **(B)** Quantitative real-time PCR was performed using RNA extracted from young leaves of the WT and transgenic plants expressing KST::HXK1 designated GCHXK (*n* = 5). *β-actin* was used for normalization. Data are means ± SE. ND, not detected.

The potential use of the KST1 promoter to drive guard-cell expression in citrus plants was examined using transgenic citrus plants that had been transformed with *KSTpro::GFP*, in which GFP served as a reporter gene (**Figure 1A**). These plants were referred to as GCGFP plants, with GC standing for guard cells. Unlike tomato and *Arabidopsis*, in which the activity of *KST1pro* was shown to be specific to guard cells (Kelly et al., 2013), transgenic GCGFP citrus plants displayed exclusive or preferred expression in guard cells that was associated with the leaf developmental stage (**Figure 1A**). Exclusive expression of GFP was observed in guard cells of young leaves (less than 1 month old; **Figure 1A4–6**). Meanwhile, in mature leaves (more than 1 month old; **Figure 1A7–9**), expression of GFP was observed primarily in guard cells, with a very small amount of expression in epidermal pavement cells (**Figure 1A8**, blue arrows). No expression was observed in any other tissues or plant parts such as mesophyll cells (**Figure 1A6,9**) or roots (not shown), indicating that the KST1 promoter might be an efficient tool for driving guard cell expression in citrus plants. We then created transgenic citrus plants that express the *Arabidopsis* HXK1 (*AtHXK1*) under the KST1 promoter. These plants were referred to as GCHXK plants, with GC standing for guard cells. The expression of *AtHXK1* in GCHXK plants was verified using quantitative real-time PCR (**Figure 1B**). The expression of *AtHXK1* seems low relative to the reference gene actin, perhaps because actin is expressed in all types of cells. Nevertheless, this result confirms that *AtHXK1* is expressed in GCHXK plants.

### Expression of *AtHXK1* in Citrus Guard Cells Reduces Stomatal Conductance and Transpiration and Increases *WUEi*

**FIGURE 2:**
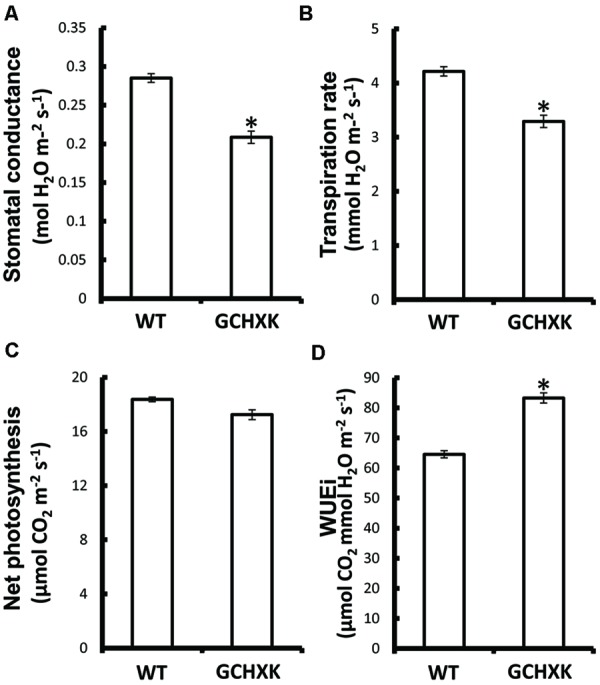
**Expression of *AtHXK1* in guard cells of citrus plants reduced stomatal conductance and transpiration with no negative effects on the rate of photosynthesis.** WT and GCHXK plants were analyzed using the LI-COR 6400 gas-exchange measurement system. **(A)** Stomatal conductance. **(B)** Transpiration rate. **(C)** Net photosynthesis. **(D)** Intrinsic water-use efficiency (*WUEi*). Data are given as means (±SE) of 8 and 11 independent repeats for the WT and GCHXK lines, respectively. Asterisks denote significant differences relative to the WT (*t*-test, *P* < 0.01).

**FIGURE 3:**
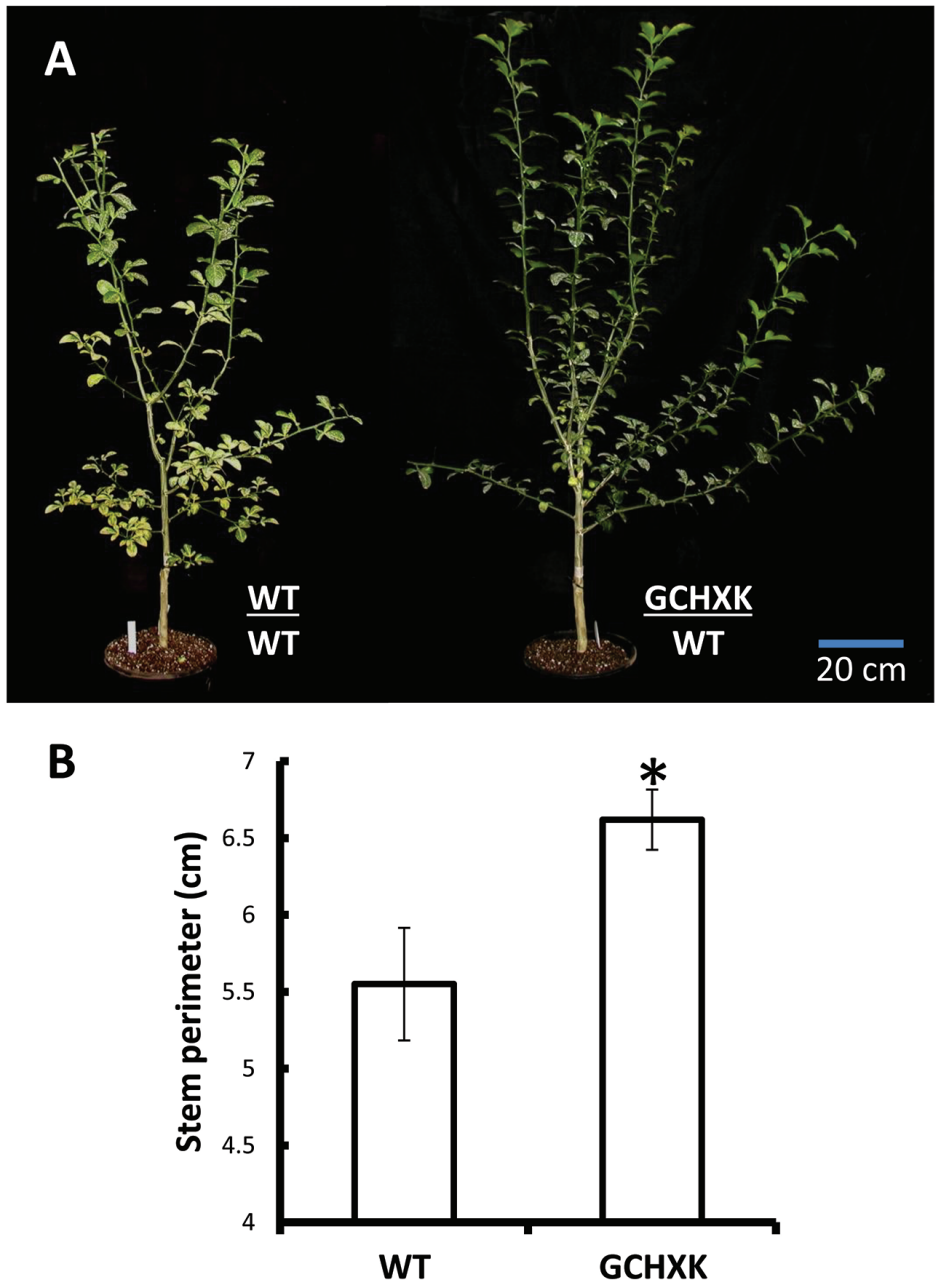
**Expression of *AtHXK1* in the guard cells of citrus plants improves plant growth. (A)** Representative images of wild-type Troyer citrange and GCHXK transgenic line expressing *AtHXK1* specifically in guard cells, both grafted on Troyer citrange rootstocks. The grafts of these plants were performed on the same day using chip buds taken from GCHXK and WT plants. **(B)** Stem diameter just above the grafting point. Data are given as means (±SE) of 4 and 5 independent repeats for the WT and GCHXK lines, respectively, 15 months after grafting. Asterisks denote significant differences relative to the WT (*t*-test, *P* < 0.05).

GCHXK and WT plants were propagated by grafting transgenic or WT shoot parts onto WT scions and the grafted plants were analyzed using the LI-COR 6400 gas-exchange system. While net photosynthesis (*A*_N_) remained unaffected (**Figure 2C**), the stomatal conductance (*g*_s_) and transpiration of the GCHXK plants were reduced (**Figures 2A,B**), leading to increased intrinsic water-use efficiency (*WUEi*; **Figure 2D**), calculated as the ratio of *A*_N_/*g*_s_ (Gago et al., 2014). No differences in stomatal density or specific leaf area were observed between WT and GCHXK leaves (Supplementary Tables S2 and S3), indicating that the lower stomatal conductance and transpiration could not be attributed to changes in leaf morphology (Gago et al., 2014). Following the increase in *WUEi*, we wished to examine the growth of the GCHXK plants. The growth of the GCHXK plants seemed to be slightly enhanced over several months (**Figure 3A**). To avoid destructive measurements, we measured the perimeter of the stem just above the grafting point (as a parameter of growth). GCHXK plants had significantly wider stems 15 months after grafting (**Figure 3B**), indicating enhanced growth of GCHXK plants.

### The Effect of *AtHXK1* on the Whole-Plant Transpiration Rate

**FIGURE 4:**
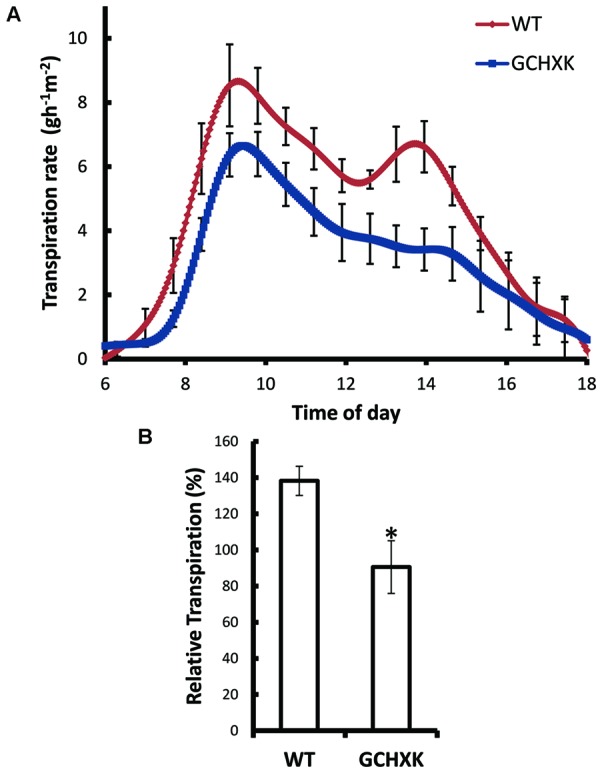
**Guard-cell expression of *AtHXK1* reduces the rate of transpiration. (A)** Transpiration rates of the WT (red line), GCHXK (blue line) plants were monitored continuously throughout the day. The rate of transpiration was normalized to the total leaf area and the amount of water taken up by the neighboring submerged fixed-size wick each day, which was set to 100%. **(B)** Whole-plant average relative daily transpiration per unit leaf area of the WT and GCHXK plants. Data points are means ± SE (*n* = 4 for WT, *n* = 4 for GCHXK). The asterisk denotes a significant difference relative to the WT (*t*-test; *P* < 0.01).

The effect of *AtHXK1* on the transpiration rate was further examined with intact grafted GCHXK and WT plants using a precise and sensitive lysimeter-scale system (Sade et al., 2010; Wallach et al., 2010). Continuous measurement of the rate of transpiration over the course of the day revealed that the transpiration rate per unit leaf area was significantly reduced in GCHXK plants (**Figure 4A**) and that the cumulative whole-plant relative daily transpiration per unit leaf area (RDT) was reduced accordingly (**Figure 4B**). The transpiration rate of GCHXK was notably lower in the middle of the day (between 9:00 a.m. and 3:00 p.m.) (**Figure 4A**), when transpiration increases (Ribeiro and Machado, 2007) and more sucrose is supposedly being carried toward the guard cells. These results suggest that HXK plays a role in the regulation of citrus stomatal aperture over the course of the day, stimulating stomatal closure, probably in response to sugar levels.

### New Rooted-Leaves System to Analyze GCHXK and WT Leaves

**FIGURE 5:**
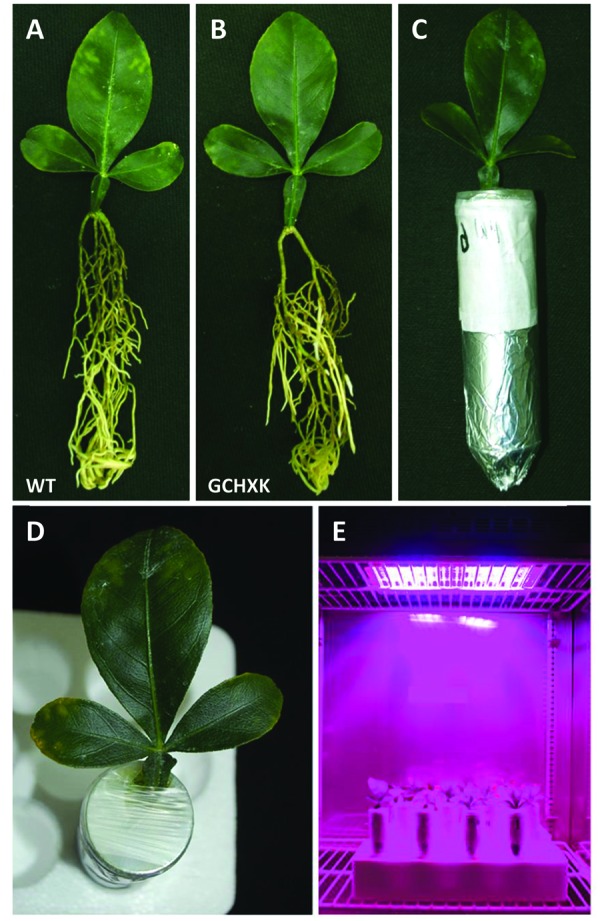
**A new rooted-leaves model system. (A)** Rooted WT citrus leaf. **(B)** Rooted GCHXK leaf. **(C)** Rooted leaves planted in a 50-ml Falcon tube filled with perlite. **(D)** The tube around the leaf was covered with Parafilm to reduce evaporation. **(E)** Rooted WT and rooted GCHXK leaves were placed in a growth chamber with controlled temperature, humidity and lighting.

**FIGURE 6:**
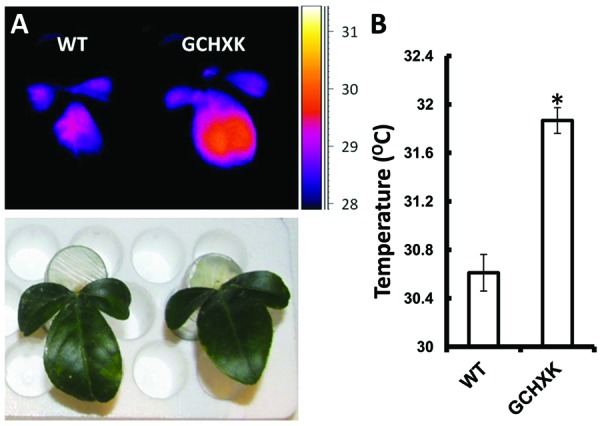
**Guard-cell expression of *AtHXK1* increases leaf temperature. (A)** Images of rooted leaves under a light intensity of 600 μmol/m^2^⋅s were captured using a thermal camera (ThermaCAM model SC655; FLIR Systems); warm colors represent high temperatures (scale is shown at right). **(B)** Leaf temperatures of the WT and GCHXK lines determined using ThermaCAM researcher pro 2.10 software. Data are means ± SE from 9 and 12 biological repeats of WT and GCHXK, respectively. The asterisk denotes a significant difference relative to the WT (*t*-test; *P* < 0.01).

To further study the effect of increased expression of *AtHXK1* in citrus guard cells, we developed a new rooted-leaves system that allowed us perform physiological experiments under controlled stable conditions (for detailed description of the method please see the “Rooting leaves” subsection of the Material and Methods). Leaves of WT and GCHXK plants were rooted (**Figures 5A,B**) and the rooted leaves were planted in 50-ml plastic tubes filled with perlite, which were covered with Parafilm to reduce water evaporation (**Figures 5C,D**). The rooted leaves were placed in a temperature-controlled growth chamber, which provided adjustable uniform growth conditions such as temperature and light intensity (**Figure 5E**). Since reduced transpiration is known to increase leaf temperature (Merlot et al., 2002), we used infrared thermal imaging to compare the temperatures of WT and GCHXK leaves (**Figure 6A**). The temperatures of the GCHXK leaves were significantly higher than those of the WT leaves (**Figure 6B**), indicating a reduced rate of transpiration in rooted GCHXK leaves.

**FIGURE 7:**
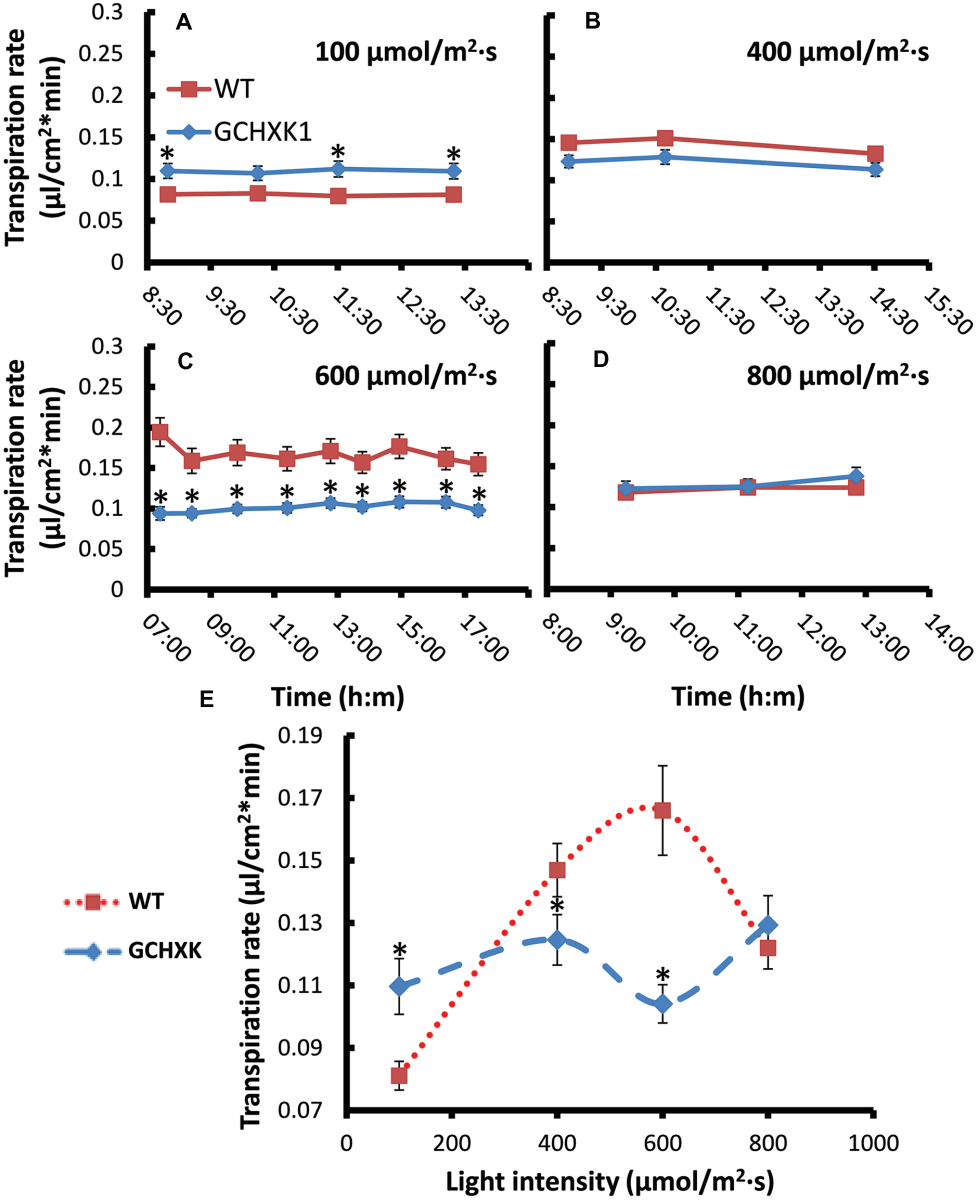
**Transpiration rates of WT and GCHXK leaves under different light intensities. (A–D)** Transpiration rate per unit leaf area of rooted WT and rooted GCHXK leaves at different light intensities **(A)** 100 μmol/m^2^ s. **(B)** 400 μmol/m^2^ s. **(C)** 600 μmol/m^2^ s **(D)** 800 μmol/m^2^ s.measured throughout the day. The transpiration rate per unit leaf area was calculated based on the weight loss noted between two consecutive measurements divided by leaf area. Data are means ± SE from at least 11 and 13 repeats of WT and GCHXK, respectively. **(E)** A plot of the mean transpiration rates versus light intensity. Data are daily means ± SE from at least 11 and 13 repeats of WT and GCHXK, respectively. The asterisks denote significant differences relative to the WT (*t*-test; *P* < 0.01).

The stomatal-closure effect of HXK is thought to be dependent on the amount of sugar produced through photosynthesis. Accordingly, stomata closure and transpiration might be affected by light intensity. We, therefore, used the rooted-leaf system to follow the rate of transpiration at various light intensities with the assumption that, at optimal light-intensity levels, GCHXK leaves may exhibit a lower rate of transpiration than WT leaves. The transpiration rates of GCHXK and WT leaves were measured at various light intensities (100, 400, 600, and 800 μmol/m2 s) by consecutively weighing the tubes throughout the day. It appeared that the transpiration rate at each light intensity remained quite consistent over the course of the day (**Figures 7A–D**), perhaps due to the constant conditions within the growth chamber. At low light intensity (100 μmol/m^2^⋅s), the transpiration rate of the rooted GCHXK leaves was slightly higher than that of the WT rooted leaves and, at 800 μmol/m2⋅s, it was similar to that of the WT. Yet, at 400 μmol/m^2^⋅s, the transpiration rate of the GCHXK rooted leaves was slightly, but significantly lower than that of the WT leaves and at 600 μmol/m2⋅s, [considered an optimal light intensity for citrus (Pimentel et al., 2004)], the transpiration rate of the rooted GCHXK leaves was half that of the WT (**Figure 7C**), indicating a significant reduction in stomatal aperture. The constant transpiration rate at each light intensity allowed us to plot the mean transpiration rate at each light intensity versus light intensity (**Figure 7E**). While the transpiration rate of the WT leaves was significantly affected by light intensity, being very low under low light-intensity conditions and peaking at 600 μmol/m^2^⋅s, the transpiration rates of the GCHXK leaves were relatively constant at the various light intensities, with the lowest transpiration rate noted at 600 μmol/m^2^⋅s. These results suggest that HXK moderates the stomatal response to light intensity and may even increase stomatal opening at low light intensities.

### The Effect of Humidity on the Transpiration Rate of GCHXK Leaves

**FIGURE 8:**
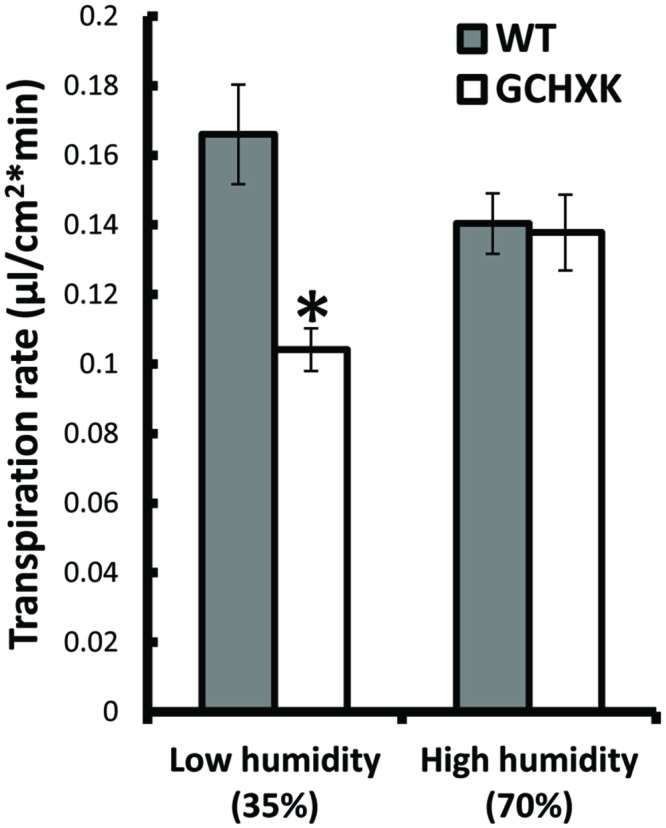
**The effect of humidity on the transpiration rate of GCHXK leaves.** Mean daily transpiration rates of WT and GCHXK leaves were measured under low-humidity (35%) and high-humidity (70%) conditions. Data are means ± SE from 8 biological repeats of WT and GCHXK leaves. The asterisks denote significant differences relative to the WT at low-humidity (*t*-test; *P* < 0.01).

It is assumed that HXK within guard cells stimulates stomatal closure in response to the amounts of sugars that are carried to guard cells by the transpiration stream. We hypothesized that under low-humidity conditions (high VPD), the transpiration rate increases transiently and more sugars are carried toward the guard cells, which may enhance the closure effect in GCHXK stomata and reduce the transpiration rate. To examine this hypothesis, we exposed rooted WT and rooted GCHXK leaves to high (70%) and low (35%) levels of humidity and measured their respective transpiration rates. Under high-humidity conditions, the transpiration rates of WT and GCXK leaves were similar, but, at the low level of humidity, the transpiration rate of GCHXK was significantly lower than that of the WT (**Figure 8**). These results support our hypothesis that VPD is a central component of the stomatal-closure response mediated by HXK, such that a high transpiration rate occurring under high VPD conditions may accelerate HXK-mediated stomatal closure and reduce transpiration.

## Discussion

The current study was motivated by a previous study in which we showed that HXK within guard cells mediates stomatal closure in *Arabidopsis* and tomato (Kelly et al., 2013). The results of the current study support our hypothesis that stomatal closure is mediated by HXK within guard cells and that this is the case not only in annual plants, but in perennial plants as well. HXK is the only enzyme that can phosphorylate glucose in plants (Granot et al., 2013) and, therefore, we would expect to find it in all plant species, including citrus plants. In fact, several citrus HXK mRNAs appear in databases (Supplementary Table S1) and HXK activity has been detected in citrus leaves (Lu et al., 2014).

The observed stomatal-closure effect was stronger in citrus plants expressing *AtHXK1* in their guard cells (GCHXK plants) and the transpiration rate of intact GCHXK plants was lower than that observed in WT plants primarily in the middle of the day when light intensity and sugar production are probably high (**Figure 4**). The transpiration rate of rooted GCHXK leaves was also lower than that of rooted WT leaves at optimal light intensity, indicating a link between sugar production, HXK and stomatal closure. The link between sugar, HXK and stomatal closure could be part of a natural mechanism to coordinate photosynthesis with transpiration. It has been shown that sucrose concentrations increase in leaves over the course of the photoperiod (Blasing et al., 2005; Comparot-Moss et al., 2010). It has also been shown that, in the apoplastic loader *Vicia faba*, some of the sugar produced over the course of the photoperiod is carried by the transpiration stream and accumulates in the vicinity of the guard cells (Lu et al., 1995; Outlaw and De Vlieghere-He, 2001). Citrus is an apoplastic loader in which sucrose produced in the mesophyll cells is exported to the apoplast prior to being loaded into the phloem (Lowell et al., 1989; Etxeberria et al., 2005; Hijaz and Killiny, 2014). The amount of apoplastic sucrose carried by the transpiration stream and arriving at the guard cells is probably increased when the rates of photosynthesis and transpiration are high.

GCHXK plants exhibit less transpiration than WT plants, especially in the middle of the day (between 9 a.m. and 3 p.m.; **Figure 4**) when transpiration rates are high and more sucrose is supposedly being carried toward the guard cells. Our observation that low humidity also reduces the transpiration of GCHXK rooted leaves (**Figure 8**) further supports the hypothesis that sugars carried by the transpiration stream are sensed by HXK within guard cells and close stomata. At low humidity levels, the large difference in water potential between the outside and inside of the leaf (high VPD) is bound to accelerate transpiration, so that more sugar is carried toward the guard cells, which would eventually reduce the stomatal apertures and transpiration of GCHXK (Outlaw and De Vlieghere-He, 2001).

The effect of low humidity on stomatal closure and gene expression has been tested in *Arabidopsis* and, in that study, few sugar-cleaving enzymes and sugar transporters were up-regulated (Bauer et al., 2013). Based on the previous hypothesis of sugars as osmolytes that open stomata, the authors of that study suggested that low humidity accelerates the export of sugars from the guard cells, to reduce guard cell osmolarity and close stomata (Bauer et al., 2013). However, our results may suggest that sugars are imported into guard cells when the humidity level is low, which would stimulate stomatal closure.

In spite of its effects on stomatal closure, the expression of *AtHXK1* in guard cells had no negative effect on plant growth and development, but rather improved growth and increased *WUEi* (**Figures 2** and **3**; Kelly et al., 2013), indicating that the total amount of sugars produced over the course of the day was not impaired. Previous efforts to reduce water loss by manipulating the number of stomata or stomatal responses led to increased WUE, but frequently reduced growth and yield, probably due to an unbalanced reduction in CO_2_ uptake, which lowered photosynthesis (Boyer, 1982; Condon et al., 2004; Blum, 2005; Dow et al., 2014; Kim et al., 2014; Roche, 2015). We assume that in the case of GCHXK, AtHXK1 within the guard cells accelerates stomatal closure only when sugar production exceeds the plant’s phloem-loading and transport capacities (Nikinmaa et al., 2013), which would explain why no negative effect on plant growth was observed. We also suggest that a surplus of sugars may serve as a signal to close stomata, reduce CO_2_ uptake and temporarily decrease the rate of photosynthesis. When transpiration decreases and sugar levels drops, stomata re-open and the rate of photosynthesis increases, thereby balancing sugar levels with the rate of transpiration.

Sucrose must be cleaved outside or inside the guard cells to be sensed by HXK. Only two groups of enzymes can cleave sucrose in plants, invertases (INV) and sucrose synthases (SUS). Apoplastic or intracellular INV cleave sucrose into glucose and fructose while intracellular SUS cleaves sucrose into UDP-glucose and fructose. The hexose monomers, glucose and fructose, are both substrates of HXK, but the affinity of HXK to glucose is two orders of magnitude higher than its affinity to fructose (Granot, 2007). Several studies have been conducted in recent years to explore the role of sugars in stomatal gene expression and movement (Antunes et al., 2012; Bates et al., 2012; Daloso et al., 2015a,b). A few studies have suggested that sucrose metabolism contributes to stomatal opening, perhaps through energy production rather than an osmotic effect (Antunes et al., 2012; Daloso et al., 2015a,b). The findings of those studies may seem to conflict with our observation of the closure effect of sugars, yet it is important to note that those studies focused on the opening stage of stomata. Furthermore, these studies show that expression of SUS in guard cells contributes to the opening (Daloso et al., 2015a,b). SUS cleaves sucrose to fructose and UDP-glucose and yields no glucose, the preferred substrate of HXK (Granot, 2007). Nevertheless, our work does not exclude and even support the possibility that sucrose metabolism may yield energy required for the opening of stomata (see below about the effect of low light intensity).

Another study explored the effect of trehalase, an enzyme that cleaves trehalose (a glucose–glucose disaccharide) on stomatal movement, and found that increased expression of trehalase reduces stomatal aperture (Van Houtte et al., 2013). The findings of that study are in line with our observation that HXK closes stomata, since trehalose cleavage yields glucose monomers, which are the primary substrate of HXK (Granot, 2008).

The rooted-leaves system provided an accurate, controlled and easy-to-manipulate biological setup for reproducible physiological experiments under uniform, stable conditions. Once harvested from the trees, the citrus leaves did not expand any further and their size remained constant throughout the rooting period and afterward. Accordingly, the rooted-leaves set-up proved to be a reliable source of experimental data. Rooting of citrus leaves was done in the past to explore vegetative propagation following the cutting of the midribs of lemon leaves. In those cases, roots were regenerated from the cut and shoot regeneration was sometimes observed (Salomon and Mendel, 1964). However, to the best of our knowledge, no further use was made of those rooted leaves (Salomon and Mendel, 1964).

The consistent conditions in the growth chamber (temperature, humidity, and light intensity) are probably the reason for the fairly consistent transpiration rates of the WT and GCHXK over the course of the day. Yet, the rooted GCHXK leaves were less affected by light intensity than the WT leaves. While at low light intensity (100 μmol/m^2^⋅s), the transpiration of GCHXK was higher than that of the WT rooted leaves, at the optimal light intensity of 600 μmol/m^2^⋅s (Pimentel et al., 2004), the transpiration rate of GCHXK was significantly lower than that of the WT (**Figure 7**). Stomatal opening is energy-dependent and requires the activation of proton ATPases (Shimazaki et al., 2007). We, therefore, assume that at a low light intensity, ATP generated from glucose metabolism following the phosphorylation of glucose by HXK may provide energy that accelerates the opening of stomata of GCHXK leaves, in line with other studies that have suggested that sucrose metabolism provides energy for the opening of stomata (Daloso et al., 2015a,b). At a high light intensity, on the other hand, the excess of sugar might be sensed by HXK and stimulate stomatal closure. Thus, HXK may have both opening and closing functions, which are dependent on the level of sugars.

Two different growth strategies have generally been associated with woody (perennial) and non-woody (annual) plants. Annual plants have higher rates of photosynthesis and higher stomatal conductance and *WUEi*, perhaps to accommodate their short life spans (Gago et al., 2014). In that regard, it could have been thought that perennials might be less sensitive to stomatal regulation by sugars and HXK. Yet, studies with robusta coffee (*Coffea canephora* – a perennial plant) have shown that shade (low light intensity) increases stomatal conductance (*g*_s_) and high light intensity reduces (*g*_s_), supporting our hypothesis that sugars might regulate and reduce stomatal aperture also in perennial species (Rodríguez-López et al., 2013). No studies dealing with direct effects of sugars on perennial stomata could be found and this is probably the first study to show that HXK stimulates stomatal closure in trees.

## Conclusion

The scientific understanding of the role of sugars in the regulation of guard-cell behavior has been revised over the course of the last century. Originally, sugars were considered the major osmolytes that open stomata and recent studies suggest that sugar metabolism contributes to stomatal opening. Yet, our study and others have shown that sugar stimulates stomatal closure, thereby coordinating photosynthesis with transpiration (Kelly et al., 2013; Lawson et al., 2014; Li et al., 2015). A previous study demonstrated that HXK within guard cells mediates stomatal closure in the annual species *Arabidopsis* and tomato. The current study shows that HXK also mediates stomatal closure in citrus trees, suggesting that this might be a widespread mechanism for coordinating photosynthesis with transpiration.

## Author Contributions

NL, GK, ER, NC, and DG planned and designed the research. NL, GK, and DG wrote the manuscript. NL, GK, LF, YY, ZA, and AL performed experiments. NL, GK, AL, VA, MM, ER, NC and DG analyzed the data.

## Conflict of Interest Statement

The authors declare that the research was conducted in the absence of any commercial or financial relationships that could be construed as a potential conflict of interest.
