# Supplementary material for: Expression of Arabidopsis Hexokinase in Citrus Guard Cells Controls Stomatal Aperture and Reduces Transpiration
Source: Front Plant Sci. 2015 Dec 16;6:1114. doi: 10.3389/fpls.2015.01114 (PMC4679854; doi:10.3389/fpls.2015.01114)
Supplement: Supplementary file 1 [file Data_Sheet_1.docx]

|  | **Table S1 \| Citrus HXK mRNA** |
| --- | --- |
| Accession no. | Description |
| NM_001288857 | *Citrus sinensis* hexokinase (LOC102577960), mRNA |
| XM_006473586 | PREDICTED: *Citrus sinensis* hexokinase-2, chloroplastic-like (LOC102626483), mRNA |
| XM_006465638 | PREDICTED: *Citrus sinensis* hexokinase-1-like (LOC102612701), mRNA |
| XM_006466326.1 | PREDICTED: *Citrus sinensis* probable hexokinase-like 2 protein-like (LOC102607776), mRNA |
| XM_006470637 | PREDICTED: *Citrus sinensis* hexokinase-3-like (LOC102626762), mRNA |

| **Table S2 \|Specific leaf area of WT and GCHXK leaves** | | |
| --- | --- | --- |
| GCHXK | WT | Line |
| 0.0708 ± 0.00576 | 0.0699 ± 0.00366 | Specific leaf area (cm^2^/mg) |

Data are mean specific leaf areas of 12 independent repeats of WT and GCHXK ± SE. Leaf area was measured using ImageJ software (<http://rsb.info.nih.gov/ij/>). Leaves were dehydrated for 3 days at 60^o^C. Specific leaf area was calculated as the ratio of leaf area to leaf dry mass.

| **Table S3 \| Stomatal density in WT and GCHXK leaves** | | |
| --- | --- | --- |
| GCHXK | WT | Line |
| 439.4 ± 11.51 | 441.7 ± 6.03 | Stomatal density (per mm^2^) |

Data are mean numbers of stomata per 1 mm^2^ leaf area, 3 samples of 0.12mm^2^ per leaf from 5 leaves of WT and GCHXK ± SE.
